# One-Pot Synthesized Biomass C-Si Nanocomposites as an Anodic Material for High-Performance Sodium-Ion Battery

**DOI:** 10.3390/nano10091728

**Published:** 2020-08-31

**Authors:** Sankar Sekar, Abu Talha Aqueel Ahmed, Deuk Young Kim, Sejoon Lee

**Affiliations:** 1 Division of Physics & Semiconductor Science, Dongguk University-Seoul, Seoul 04620, Korea; sanssekar@gmail.com (S.S.); talhaphy@gmail.com (A.T.A.A.); dykim@dongguk.edu (D.Y.K.); 2 Quantum-functional Semiconductor Research Center, Dongguk University-Seoul, Seoul 04620, Korea

**Keywords:** silicon, activated carbon, nanocomposite, sodium ion battery, biomass

## Abstract

Aiming at materializing an excellent anodic source material of the high-performance sodium-ion battery (SIB), we fabricated the biomass carbon-silicon (C-Si) nanocomposites by the one-pot synthesis of facile magnesiothermic reduction using brown rice husk ashes. The C-Si nanocomposites displayed an aggregated morphology, where the spherical Si nanoparticles (9 nm on average) and the C nanoflakes were encapsulated and decorated with each other. When utilizing the nanocomposites as an SIB anode, a high initial discharge capacity (i.e., 378 mAh/g at 100 mA/g) and a high reversible capacity (i.e., 122 mAh/g at 200 mA/g) were achieved owing to their enhanced electronic and ionic conductivities. Moreover, the SIB device exhibited a high cyclic stability in its Coulombic efficiency (i.e., 98% after 100 charge-discharge cycles at 200 mA/g). These outstanding results depict that the one-pot synthesized biomass C-Si nanocomposites are beneficial for future green energy-storage technology.

## 1. Introduction

For next-generation green energy-storage device technology, the sodium-ion battery (SIB) has emerged as a favorable alternative to the lithium-ion battery (LIB) because of its huge abundance, low cost, eco-friendliness, non-toxicity, and analogous electrochemical mechanisms [1,2,3]. Despite the similarity in their electrochemical operation mechanisms of both LIBs and SIBs, however, the electrode materials of LIBs are incompatible with those of SIBs because of different ionic radii between the sodium ion (Na^+^: 0.102 nm) and the lithium ion (Li^+^: 0.076 nm) [4]. For instance, although crystalline graphite is adequate as a commercial LIB anode [5,6], the weak Na^+^ intercalation into graphite [7,8] makes it inappropriate as an SIB anode [9]. Therefore, developing an excellent anode material is essential for demonstrating high-performance SIBs. Accordingly, several types of SIB anode materials have been proposed in resent studies [10,11,12,13,14]; for instance, tin (Sb) [15], antimony [16], phosphorus (P) [17], porous carbon (C) [18], silicon (Si) [19], titanium dioxide (TiO_2_) [20], and chalcogenides [21] are typical examples that can move a step closer to the practical application of SIBs. Among them, Si is one of the most distinctive anode materials for rechargeable batteries because of theoretical expectations on its high specific capacity (i.e., 4200 mAh/g for LIBs [5] and 954 mAh/g for SIBs [22,23]). In many experimental attempts [23,24,25], however, the Si anode revealed the poor reversible discharge capacity because of both the formation of metastable NaSi crystallites and the volumetric change of Si matrix during the sodiation-desodiation processes. To avoid this, recently, various Si nanoarchitectures and Si-C nanocomposites (e.g., Si microspheres [19], Si nanocrystals [25], Si nanowires [26], Si nanoparticles [27], amorphous Si [28], SiO film [29], Si/C nanocomposites [30], Si/SiO_2_/mesoporous-C nanocomposites [31], etc.) have been widely studied in many experiments. In addition, a variety of the synthesis method was proposed for the fabrication of the high-quality porous Si and SiO_2_ nanostructures. For example, wet etching [32], chemical doping [33], laser ablation [34], chemical vapor deposition [35], and magnesiothermic reduction [36,37] are feasible ways that can yield the high-quality siliceous nanostructures. Despite such substantial efforts, the reversible discharge capacity still remains ineffectual because the large portion of irreversible Na^+^ ions would tend to reside in Si matrix during the desodiation process [27]. Furthermore, high cost and high level of difficulty for synthesizing the Si nanostructures may also restrict their tangible applications for the SIB anodes. In consideration of both the cost-effectiveness and the simplicity, compared to others above, the magnesiothermic reduction process is one of the most facile and cheap techniques because magnesiothermic reaction could perform at low temperature in inert atmosphere with no vacuum facilities and toxic gases. Considering also the environmental friendliness, meanwhile, the biomass rice husk is one of the most abundant natural resources that can supply various kinds of carbonaceous and siliceous sources [38,39]. Upon such benefits, a lot of Si and C nanostructures were derived from rice husks for the LIB and the SIB applications; e.g., graphene [39], porous carbon [40], activated carbon [41], zeolites [42], silicon carbide [43], silica [44,45,46], silicon tetrachloride [47], silicon nitride [48], silicon nanocrystals [36,49], etc. In spite of such an availability for both carbonaceous and siliceous natures from rice husks, however, the simultaneous derivation of the C-Si nanocomposites has rarely been investigated, except for few previous works [36,38]. To our best survey, furthermore, no studies have been conducted yet in order for the utilization of the biomass C-Si nanocomposites as a SIB anode.

Motivated by all the above, we fabricated the C-Si nanocomposites by the one-pot synthesis method of facile magnesiothermic reduction using biomass brown rice husk (BRH) ashes, and assessed their electrochemical characteristics as a SIB anode. The fabricated SIB device with a biomass C-Si nanocomposite anode showed the excellent energy-storage capacity, outstanding cycle stability, and good rate performance. Herein, we report experimental data on the synthesis-to-device application of the C-Si nanocomposites in detail.

## 2. Experimental Section

### 2.1. Materials Preparation

The magnesium (Mg, 99% purity) powder, hydrofluoric acid (HF, 48%), and hydrochloric acid (HCl, 37%) were purchased from Sigma-Aldrich (St. Louis, MO, USA), and used without further purification. The biomass resource—BRH was collected from Perambalur, Tamil Nadu, India.

### 2.2. One-Pot Synthesis of C-Si Nanocomposites

**Figure 1:**
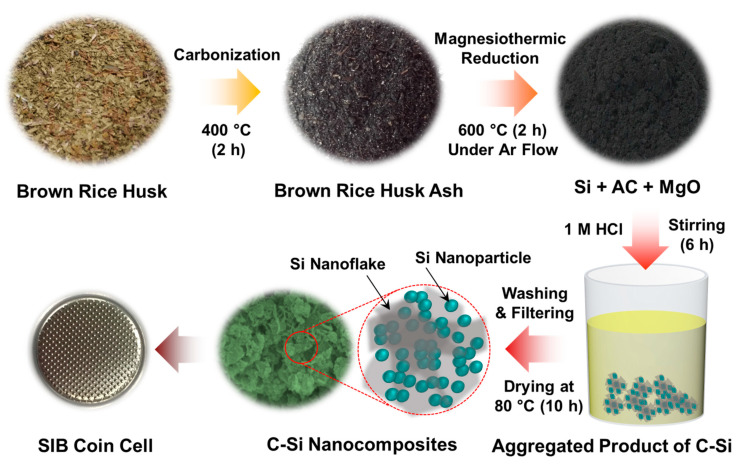
Schematic illustration for the one-pot synthesis of the C-Si nanocomposites via facile magnesiothermic reduction using BRH ashes.

Figure 1 schematically represents the one-pot synthesis of the C-Si nanocomposites through magnesiothermic reduction using BRH ashes. As an initial task, BRHs were calcinated at 400 °C for 2 h in air to collect the carbonaceous and siliceous resource of the BRH ashes. Then, we mixed the BRH ashes (1.2 g) and the Mg powder (0.3 g) using a mortar and annealed those mixtures at 600 °C for 2 h in Ar atmosphere. During the annealing process, the C-Si, MgO, and Mg_2_Si components are produced via magnesiothermic reduction and the chemical reaction described below:

(1) $$SiO_{2}/C\;\left(\mathrm{BRH}\;\mathrm{ashes}\right)+2\mathrm{Mg}\underset{600\;{}^{\circ}C}{\to }C-\mathrm{Si}+2\mathrm{MgO}.\;$$

This allows the one-pot synthesis of C-Si nanocomposites from BRH ashes via magnesiothermic reduction. Next, to remove residual impurities and precipitates (e.g., Mg_2_Si, unreacted SiO_2_, MgO residues, etc.), the mixtures were stirred in HCl (1 M) for 360 min, and were subsequently soaked with hydrofluoric acid (5%) for 60 min. Finally, the powder type of the C-Si nanocomposite was obtained through three-times-repeated processes of “rinsing in deionized (DI) water→filtering→drying at 80 °C for 10 h in a vacuum”.

### 2.3. Characterization of Material Properties

The crystallographic characteristics of the C-Si nanocomposites were investigated by X-ray diffraction (XRD) using a D2 Phaser system (Bruker, Madison, WI, USA). The vibrational characteristics of the nanocomposites were analyzed by Raman scattering spectroscopy using a LabRAM HR800 system (HORIBA Jobin Yvon Inc., Edison, NJ, USA). The morphological and the compositional properties were examined by field-emission scanning electron microscopy (FE-SEM) and in-situ energy dispersive X-ray (EDX) spectroscopy, respectively, using an Inspect F50 system (FEI Company, Mahwah, NJ, USA). Additionally, the microstructures of the nanocomposites were monitored by transmission electron microscopy (TEM) and in-situ selective area electron diffraction (SAED) using a Titan 80–300 microscope (FEI Company, Hillsboro, OR, USA).

### 2.4. Fabrication of SIB Device Using C-Si Nanocomposites

The 2032 coin-cell type of the SIB device was fabricated to assess the electrochemical performances of the C-Si nanocomposites. For preparing the SIB anode, firstly, the C-Si nanocomposites (80 wt.%) were mixed with carbon black (10 wt.%) and polyvinylidene difluoride (PVDF) (10 wt.%) in n-methyl-2-pyrrolidinone. Then, the prepared slurry was coated onto the Cu foil, and was subsequently dried at 110 °C for 5 h in vacuum. Here, the mass loading of the electrode was 0.7 mg. In addition, we also note that the used electrolyte to involve a mixture of ethylene carbonate and dimethyl carbonate in 1 M NaPF_6_ solvent at a volume ratio of 1:1. Finally, the coin-cell SIB device was assembled in a glove box under Ar (99.999%) ambience. Here, we also note that sodium foil and Celgard 2400 were used as the counter electrode and as the separator, respectively.

### 2.5. Characterization of Electrochemical Performances

The electrochemical performances of the fabricated SIB device were examined through the cyclic voltammetry (CV), galvanostatic charge-discharge (GCD), and electrochemical impedance spectrometry (EIS) measurements by using a MPG2 potentiostat (BioLogic, Seyssinet-Pariset, France). The CV characteristics were tested at the potential range of 0.01–2.5 V (vs. Na/Na^+^) under the scan rate (r_s_) of 0.2 mV/s, and the GCD characteristics were examined at 0.01–2.5 V (vs. Na/Na^+^) by applying the constant current of 100–2000 mA/g. The charge-transfer impedance characteristics were measured by EIS at the frequency range from 0.01 Hz to 100 kHz.

## 3. Results and Discussion

### 3.1. Structural, Morphological, and Microstructural Properties of C-Si Nanocomposites

**Figure 2:**
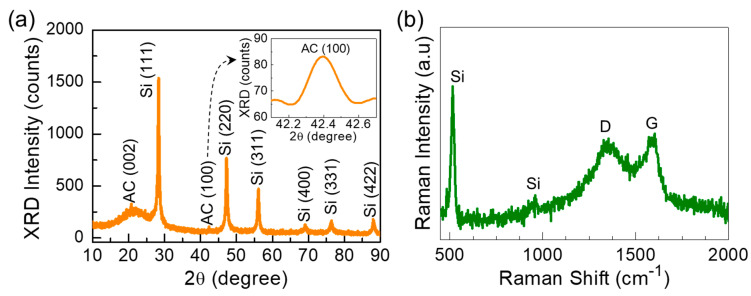
(**a**) XRD patterns and (**b**) Raman spectrum of the C-Si nanocomposites. The inset of (**a**) displays the zoom-in view of the AC (100) peak.

Figure 2a shows the XRD patterns of the C-Si nanocomposites. The sample exhibited only the diffraction patterns from the intrinsic composite species of Si and C. The six clear diffraction patterns at 28.2°, 47.1°, 56.1°, 69°, 76.4°, and 87.9° correspond to the (111), (220), (311), (400), (331), and (422) lattice planes of crystalline Si (JCPDS no.27-1402) [36,50,51], respectively; and the broad peak at 22° and the small peak at 42.4° arise from the (002) and (100) phases of activated carbon [52,53,54], respectively. Using the Scherer formula [55,56,57], the average crystal size of the C-Si nanocomposites was determined to be approximately 14 nm. From the Raman scattering spectroscopy measurement, the sample also revealed its intrinsic vibration properties from only Si and C. As displayed in Figure 2b, the sample showed the four predominant Raman bands at 514, 959, 1341, and 1590 cm^−1^ from Si and C. The former two bands at 514 and 959 cm^−1^ originate from the first and the second order transversal optical (TO) mode of crystalline Si [58,59], respectively; and the latter two vibration modes at 1341 and 1590 cm^−1^ come from D (sp^3^ type) and G (sp^2^ type) bands of graphitized C, respectively [60,61]. Here, it should be noticeable that the C nanoflakes exhibited the high intensity area ratio of I_D_/I_G_ (i.e., Asp^3^/Asp^2^ ≅ 0.99), indicative of the high graphitization (i.e., ultrathin C layers) and large amount of sp^2^ carbon exists in the composite system [39,53,60,62,63,64]. The absence of extra lattice phases and their vibrations depict that the high purity C-Si nanocomposites were well-crystallized with their intrinsic carbonaceous and siliceous resources via the one-pot synthesis of magnesiothermic reduction using biomass BRH ashes.

**Figure 3:**
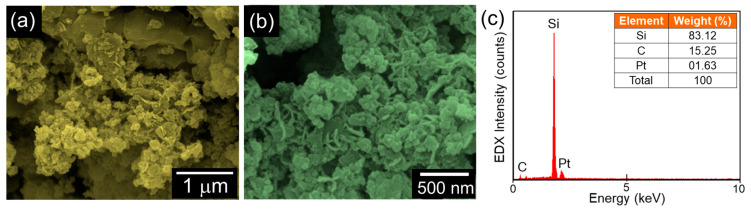
(**a**) Low-magnification FE-SEM image, (**b**) High-magnification FE-SEM image, and (**c**) EDX spectrum of the C-Si nanocomposites. The inset of (**c**) summarizes the compositional properties of the C-Si nanocomposites.

Next, the morphological properties of the C-Si nanocomposites were monitored by FE-SEM measurements. Figure 3a and b display the low- and high-magnification FE-SEM images of the C-Si nanocomposites, respectively. The sample exhibited an aggregated nanocomposite morphology, where the spherical Si nanoparticles were interconnected with the C nanoflakes. From the EDX spectrum (Figure 3c), one can also confirm that the biomass BRH-derived C-Si nanocomposites involved their intrinsic species of Si (83.12 wt.%) and C (15.25 wt.%). This corroborates that the C-Si nanocomposites were effectively synthesized from the biomass carbonaceous and siliceous resource of BRH ashes. The small portion of Pt (1.63 wt.%) is thought as sprouting from conductive-coating for the FE-SEM measurement.

**Figure 4:**
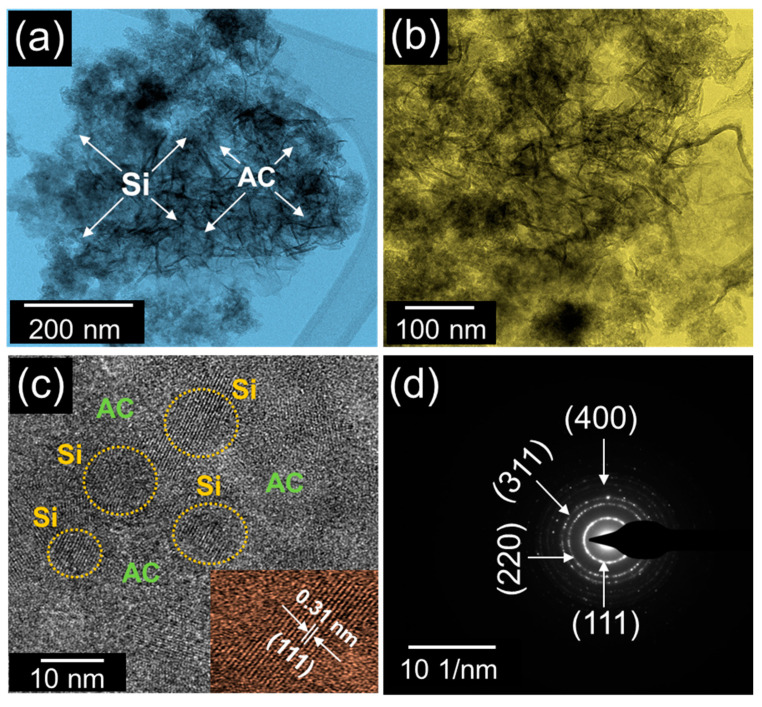
(**a**) Low-magnification TEM image, (**b**) high-magnification TEM image, (**c**) high-resolution TEM image, and (**d**) SAED patterns of the C-Si nanocomposites.

The TEM images display the further insight into the microstructures of the C-Si nanocomposites (Figure 4). As represented in high- and low-magnification TEM images, the nanocomposites were constructed in the form of the aggregated structure, consisting of the Si nanoparticles and the C nanoflakes (Figure 4a,b). Additionally, from the high-resolution TEM image, one can also observe that the spherical Si nanoparticles were entrenched with the ultrathin C nanoflakes (Figure 4c). The average size of the Si nanoparticles was determined to be 9 nm, and the interlayer spacing of the Si lattice fringes was confirmed to be 0.31 nm, corresponding to the lattice constant of crystalline (111) Si. The SAED patterns of the C-Si nanocomposites further elucidate the effective aggregation of well-crystallized Si nanoparticles with well-graphitized C nanoflakes.

### 3.2. Electrochemical Performances of C-Si Nanocomposites as an SIB Anode

**Figure 5:**
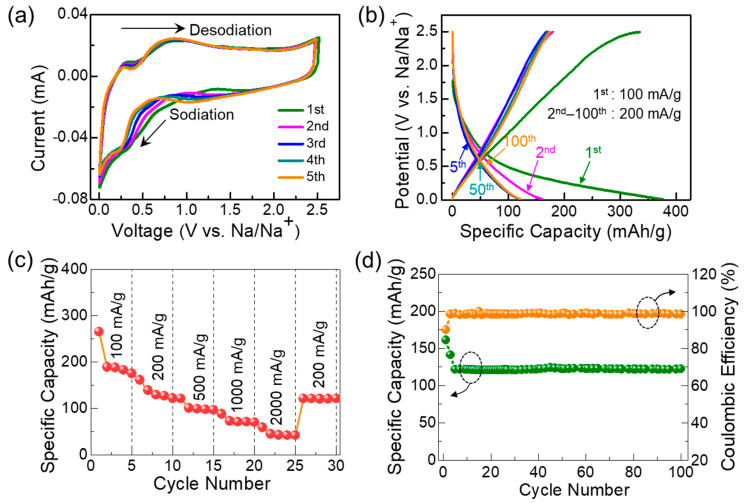
Electrochemical SIB anode performances of the C-Si nanocomposites: (**a**) CV curves measured at r_s_ = 0.2 mV/s, (**b**) GCD profiles measured under the current injection of 100–200 mA/g, (**c**) Rate performances at various current injection conditions, and (**d**) Coulombic efficiency and cyclic stability measured under the current injection of 200 mA/g.

The conformation of the C-Si nanocomposite system would be beneficial for improving the SIB anode performances because of the synergetic effects from the Si nanoparticles (i.e., high specific capacity and low discharge potential) and the C nanoflakes (i.e., high electrical conductivity and large surface area) [36,38,65]. We, therefore, assessed the electrochemical performance of the C-Si nanocomposites as aa SIB anode. Figure 5a shows the CV curves of the fabricated SIB device that comprised the C-Si nanocomposite anode. Here, the CV characteristics were measured at the potential range of 0–2.5 V (vs. Na/Na^+^) under r_s_ = 0.2 mV/s. The device clearly exhibited the charge-discharge characteristics at the potential range below 1 V. For the first CV sweep, the sample revealed a wide reduction in the sodiation regime because of the formation of the solid electrolyte interphase (SEI) layer. After the formation of the SEI layer, however, the CV curve became stable with maintaining a distinctive area. These indicate that the sodiation-desodiation effectively took place in the C-Si nanocomposite SIB anode [28,66].

The anodic performances of the C-Si nanocomposites were further examined by GCD measurements. As shown in Figure 5b, the SIB device clearly revealed the charge-discharge characteristics in its GCD curves. For the first cycle, the specific charge capacity and the discharge capacity were obtained to be 334 and 378 mAh/g, respectively, under the injection current density of 100 mA/g. This represents the C-Si nanocomposites to hold a high initial Coulombic efficiency up to 88%, attributing to the encapsulation of Si nanoparticles with C nanoflakes. Namely, since the ultrathin C nanoflakes covered the surface of the Si nanoparticles, the large number of Na^+^ ions could be intercalated and de-intercalated with the C-Si composites. Because of the capacity loss due to the inevitable formation of NaSi [14,19,30], the discharge capacity was decreased to 161 mAh/g at the 2nd cycle under the current injection of 200 mA/g. From the 5th to 100th cycles, however, the discharge capacity became stable at 122 mAh/g.

Next, we tested the rate performance and the cyclic stability of the SIB device. As represented in Figure 5c, the device showed the reversible discharge capacity of 265, 122, 101, 88, and 59 mAh/g when the injection current density was 100, 200, 500, 1000, and 2000 mA/g, respectively. Here, one can observe that the discharge capacity was effectively recovered to the specific value at the certain current injection conditions. Namely, the magnitude of the specific discharge capacity is nearly identical at both the beginning stage and the final stage under the current injection of 200 mA/g. Such a superb reversibility is also associated to the cyclic stability. As aforementioned, due to the phase transition period (i.e., SEI formation) [19], the device lost 13% of the Coulombic efficiency during the initial three cycles (Figure 5d). After the initialization, however, the SIB device maintained its high Coulombic efficiency up to 98% even after 100 cycles under the current injection of 200 mA/g. Such an excellent cyclic stability is comparable to literature values, and is even greater than other Si-based SIB anodes that were fabricated with commercial resources [19,25,26,27,28,29,30,31] (See Table 1).

**Figure 6:**
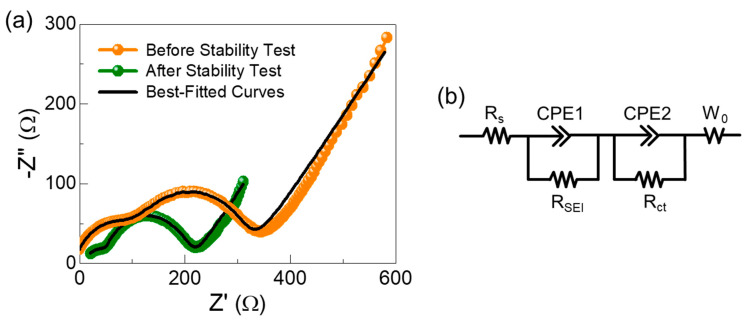
Charge-transfer characteristics of the C-Si nanocomposites: (**a**) Nyquist plots before and after the cyclic stability test and (**b**) equivalent circuit of the SIB device composed of the C-Si nanocomposites as an anodic material.

Finally, we discuss the charge-transfer characteristics of the C-Si nanocomposites as a SIB anode. As shown in the Nyquist plots (Figure 6a), the sample displayed both semicircles and long tails at low and high frequency regions, respectively. According to the equivalent circuit model [67], the former ones are attributed to the charge transfer resistance (R_ct_) [28], and the latter ones are associated with the Warburg impedance (W_0_), corresponding to Na^+^ intercalation into the electrode material [8]. Through adopting this model to our SIB device structure, we established the equivalent circuit model (Figure 6b) and fitted the experimental data to the circuit simulator to estimate the key device parameters. We, here, note that two additional resistance components of R_s_ and R_SEI_ in the equivalent circuit are the electrolyte solution resistance and the SEI resistance, respectively. CPE1 and CPE2 means the constant phase elements of the SEI layer and the electrochemical double layer in the composite, respectively [67]. Using the established equivalent circuit model, R_SEI_ and R_ct_ were determined to be 134 and 206 Ω, respectively, at the first cycle (i.e., before cyclic stability test). However, after the cyclic stability test (i.e., 100 charge-discharge cycles at 200 mA/g), R_SEI_ and R_ct_ were decreased to 79 and 174 Ω, respectively, because of the SEI formation. These low R_SEI_ and R_ct_ values infer that the C-Si nanocomposites possess high ionic and electronic conductivities, which can enhance the sodium ion intercalation into the SIB anode material via the electrode/electrolyte interface. Consequently, the excellent SIB anode performances could be thought as resulting from the formation of the C-Si nanocomposite solid-state system. In other words, the encapsulation of Si nanoparticles (i.e., high charge capacity resource) with C nanoflakes (i.e., high conductivity) could help enhance the intercalation/de-intercalation of Na^+^ ion with the anodic material of the C-Si nanocomposites.

## 4. Summary and Conclusions

The biomass C-Si nanocomposites were synthesized by the one-pot synthesis method through the facile magnesiothermic reduction process using the BRH ashes. The nanocomposites showed an interconnected and aggregated morphology, where the spherical Si nanoparticles were densely packed and encapsulated with the C nanoflakes. For the electrochemical SIB anode characteristics, the nanocomposites exhibited the high initial discharge capacity of 378 mAh/g (at 100 mA/g) as well as the prominent reversible capacity of 122 mAh/g (at 200 mA/g). Additionally, the excellent cyclic stability (i.e., 98% capacity retention after 100 cycles at 200 mA/g) was observed from the nanocomposites. These excellent anodic performances could be attributed to the encapsulation of the highly intercalative Si nanoparticles with the highly-conductive ultrathin C nanoflakes. The results suggest that the biomass BRH-derived C-Si nanocomposites hold great potential as a high-performance SIB anode material.

## Figures and Tables

**Table 1:** Comparison of the electrochemical SIB anode performances for various Si-based nanocomposites.

Resource		Anode Materials	Specific Capacity (mAh/g)	Current Density (mA/g)	Cycle Number (#)	Capacity Retention (%)	Ref.
**Biomass**	Rice Husks	C-Si Nanocomposites	122	200	100	98	This work
**Commercial**	SiO_2_	Si Microspheres	390	100	100	-	[19]
	Si Nanoparticles	Si/C Nanocomposites	454.5	50	200	-	[25]
	SiH_4_	Si Nanowires	125	72.5	100	99	[26]
	SiH_4_	Si Nanoparticles	348	20	100	92	[27]
	Si Powder	Si/C Nanocomposites	280	100	100	-	[30]
	SiO_2_-OMC	Si/SiO_2_-OMC Nanocomposites	423	50	100	-	[31]
	SiO	SiO Film	170	50	100	77	[29]
	Si Powder	Amorphous Si Powder	150	36.25	100	70	[28]

* OMC, Ordered mesoporous carbon.

## References

[1] Bray J.M., Doswell C.L., Pavlovskaya G.E., Chen L., Kishore B., Au H., Alptekin H., Kendrick E., Titirici M.-M., Meersmann T. (2020). Operando visualisation of battery chemistry in a sodium-ion battery by 23Na magnetic resonance imaging. Nat. Commun..

[2] Hwang J.-Y., Myung S.-T., Sun Y.-K. (2017). Sodium-ion batteries: Present and future. Chem. Soc. Rev..

[3] Pan H., Hu Y.-S., Chen L. (2013). Room-temperature stationary sodium-ion batteries for large-scale electric energy storage. Energy Environ. Sci..

[4] Wang Y., Liu Y., Liu Y., Shen Q., Chen C., Qiu F., Li P., Jiao L., Qu X. (2021). Recent advances in electrospun electrode materials for sodium-ion batteries. J. Energy Chem..

[5] Scrosati B., Hassoun J., Sun Y.-K. (2011). Lithium-ion batteries. A look into the future. Energy Environ. Sci..

[6] Sankar S., Saravanan S., Ahmed A.T.A., Inamdar A.I., Im H., Lee S., Kim D.Y. (2019). Spherical activated-carbon nanoparticles derived from biomass green tea wastes for anode material of lithium-ion battery. Mater. Lett..

[7] Perveen T., Siddiq M., Shahzad N., Ihsan R., Ahmad A., Shahzad M.I. (2020). Prospects in anode materials for sodium ion batteries—A review. Renew. Sust. Energ. Rev..

[8] Chandra C., Kim J. (2018). Silicon oxycarbide produced from silicone oil for high-performance anode material in sodium ion batteries. Chem. Eng. J..

[9] Wen Y., He K., Zhu Y., Han F., Xu Y., Matsuda I., Ishii Y., Cumings J., Wang C. (2014). Expanded graphite as superior anode for sodium-ion batteries. Nat. Commun..

[10] Jamesh M.-I. (2019). Recent advances on flexible electrodes for Na-ion batteries and Li–S batteries. J. Energy Chem..

[11] Li Y., Lu Y., Zhao C., Hu Y.-S., Titirici M.-M., Li H., Huang X., Chen L. (2017). Recent advances of electrode materials for low-cost sodium-ion batteries towards practical application for grid energy storage. Energy Storage Mater..

[12] Kim S.-W., Seo D.-H., Ma X., Ceder G., Kang K. (2012). Electrode Materials for Rechargeable Sodium-Ion Batteries: Potential Alternatives to Current Lithium-Ion Batteries. Adv. Energy Mater..

[13] Shangguan H., Huang W., Engelbrekt C., Zheng X., Shen F., Xiao X., Ci L., Si P., Zhang J. (2019). Well-defined cobalt sulfide nanoparticles locked in 3D hollow nitrogen-doped carbon shells for superior lithium and sodium storage. Energy Storage Mater..

[14] Dahbi M., Yabuuchi N., Kubota K., Tokiwa K., Komaba S. (2014). Negative electrodes for Na-ion batteries. Phys. Chem. Chem. Phys..

[15] Li Z., Ding J., Mitlin D. (2015). Tin and tin compounds for sodium ion battery anodes: Phase transformations and performance. Acc. Chem. Res..

[16] He J., Wei Y., Zhai T., Li H. (2018). Antimony-based materials as promising anodes for rechargeable lithium-ion and sodium-ion batteries. Mater. Chem. Front..

[17] Ni J., Li L., Lu J. (2018). Phosphorus: An anode of choice for sodium-ion batteries. ACS Energy Lett..

[18] Wenzel S., Hara T., Janek J., Adelhelm P. (2011). Room-temperature sodium-ion batteries: Improving the rate capability of carbon anode materials by templating strategies. Energy Environ. Sci..

[19] Qiu D.-F., Ma X., Zhang J.-D., Lin Z.-X., Zhao B. (2018). Mesoporous Silicon Microspheres Produced from In Situ Magnesiothermic Reduction of Silicon Oxide for High-Performance Anode Material in Sodium-Ion Batteries. Nanoscale Res. Lett..

[20] Rubio S., Maça R.R., Aragón M.J., Cabello M., Castillo-Rodríguez M., Lavela P., Tirado J.L., Etacheri V., Ortiz G.F. (2019). Superior electrochemical performance of TiO_2_ sodium-ion battery anodes in diglyme-based electrolyte solution. J. Power Sources.

[21] Dai S., Wang L., Cao M., Zhong Z., Shen Y., Wang M. (2019). Design strategies in metal chalcogenides anode materials for high-performance sodium-ion battery. Mater. Today Energy.

[22] Chevrier V.L., Ceder G. (2011). Challenges for Na-ion negative electrodes. J. Electrochem. Soc..

[23] Chou C.-Y., Lee M., Hwang G.S. (2015). A comparative first-principles study on sodiation of silicon, germanium, and tin for sodium-ion batteries. J. Phys. Chem. C.

[24] Jung S.C., Jung D.S., Choi J.W., Han Y.-K. (2014). Atom-Level Understanding of the Sodiation Process in Silicon Anode Material. J. Phys. Chem. Lett..

[25] Zhang L., Hu X., Chen C., Guo H., Liu X., Xu G., Zhong H., Cheng S., Wu P., Meng J. (2017). In Operando Mechanism Analysis on Nanocrystalline Silicon Anode Material for Reversible and Ultrafast Sodium Storage. Adv. Mater..

[26] Jangid M.K., Lakhnot A.S., Vemulapally A., Sonia F.J., Sinha S., Dusane R.O., Mukhopadhyay A. (2018). Crystalline core/amorphous shell structured silicon nanowires offer size and structure dependent reversible Na-storage. J. Mater. Chem. A.

[27] Xu Y., Swaans E., Basak S., Zandbergen H.W., Borsa D.M., Mulder F.M. (2016). Reversible Na-Ion Uptake in Si Nanoparticles. Adv. Energy Mater..

[28] Lim C.-H., Huang T.-Y., Shao P.-S., Chien J.-H., Weng Y.-T., Huang H.-F., Hwang B.J., Wu N.-L. (2016). Experimental study on sodiation of amorphous silicon for use as sodium-ion battery anode. Electrochim. Acta.

[29] Shimizu M., Usui H., Fujiwara K., Yamane K., Sakaguchi H. (2015). Electrochemical behavior of SiO as an anode material for Na-ion battery. J. Alloys Compd..

[30] Zhao Q., Huang Y., Hu X. (2016). A Si/C nanocomposite anode by ball milling for highly reversible sodium storage. Electrochem. Commun..

[31] Zeng L., Liu R., Han L., Luo F., Chen X., Wang J., Qian Q., Chen Q., Wei M. (2018). Preparation of a Si/SiO_2_–Ordered-Mesoporous-Carbon Nanocomposite as an Anode for High-Performance Lithium-Ion and Sodium-Ion Batteries. Chem. Eur. J..

[32] Vlad A., Reddy A.L.M., Ajayan A., Singh N., Gohy J.-F., Melinte S., Ajayan P.M. (2012). Roll up nanowire battery from silicon chips. Proc. Natl. Acad. Sci. USA.

[33] Ge M., Rong J., Fang X., Zhang A., Lu Y., Zhou C. (2013). Scalable preparation of porous silicon nanoparticles and their application for lithium-ion battery anodes. Nano Res..

[34] Morales A.M., Lieber C.M. (1998). A laser ablation method for the synthesis of crystalline semiconductor nanowires. Science.

[35] Yang R., Buonassisi T., Gleason K.K. (2013). Organic vapor passivation of silicon at room temperature. Adv. Mater..

[36] Sekar S., Aqueel Ahmed A.T., Inamdar A.I., Lee Y., Im H., Kim D.Y., Lee S. (2019). Activated carbon-decorated spherical silicon nanocrystal composites synchronously-derived from rice husks for anodic source of lithium-ion battery. Nanomaterials.

[37] Entwistle J., Rennie A., Patwardhan S. (2018). A review of magnesiothermic reduction of silica to porous silicon for lithium-ion battery applications and beyond. J. Mater. Chem. A.

[38] Yu K., Zhang H., Qi H., Gao X., Liang J., Liang C. (2018). Rice husk as the source of silicon/carbon anode material and stable electrochemical performance. ChemistrySelect.

[39] Sankar S., Lee H., Jung H., Kim A., Aqueel Ahmed A.T., Inamdar A.I., Kim H., Lee S., Im H., Kim D.Y. (2017). Ultrathin graphene nanosheets derived from rice husks for sustainable supercapacitor electrodes. New J. Chem..

[40] Rybarczyk M.K., Peng H.-J., Tang C., Lieder M., Zhang Q., Titirici M.-M. (2016). Porous carbon derived from rice husks as sustainable bioresources: Insights into the role of micro-/mesoporous hierarchy in hosting active species for lithium–sulphur batteries. Green Chem..

[41] Le Van K., Luong Thi T.T. (2014). Activated carbon derived from rice husk by NaOH activation and its application in supercapacitor. Prog. Nat. Sci. Mater. Int..

[42] Pereira M.M., Gomes E.S., Silva A.V., Pinar A.B., Willinger M.-G., Shanmugam S., Chizallet C., Laugel G., Losch P., Louis B. (2018). Biomass-mediated ZSM-5 zeolite synthesis: When self-assembly allows to cross the Si/Al lower limit. Chem. Sci..

[43] Sujirote K., Leangsuwan P. (2003). Silicon carbide formation from pretreated rice husks. J. Mater. Sci..

[44] Sankar S., Kaur N., Lee S., Kim D.Y. (2018). Rapid sonochemical synthesis of spherical silica nanoparticles derived from brown rice husk. Ceram. Int..

[45] Liu Y., Guo Y., Gao W., Wang Z., Ma Y., Wang Z. (2012). Simultaneous preparation of silica and activated carbon from rice husk ash. J. Clean. Prod..

[46] Sankar S., Sharma S.K., Kaur N., Lee B., Kim D.Y., Lee S., Jung H. (2016). Biogenerated silica nanoparticles synthesized from sticky, red, and brown rice husk ashes by a chemical method. Ceram. Int..

[47] Basu P.K., King C.J., Lynn S. (1973). Manufacture of silicon tetrachloride from rice hulls. AIChE J..

[48] Pavarajarn V., Precharyutasin R., Praserthdam P. (2010). Synthesis of silicon nitride fibers by the carbothermal reduction and nitridation of rice husk ash. J. Am. Ceram. Soc..

[49] Marchal J.C., Krug Iii D.J., McDonnell P., Sun K., Laine R.M. (2015). A low cost, low energy route to solar grade silicon from rice hull ash (RHA), a sustainable source. Green Chem..

[50] Kim J.M., Guccini V., Seong K.-d., Oh J., Salazar-Alvarez G., Piao Y. (2017). Extensively interconnected silicon nanoparticles via carbon network derived from ultrathin cellulose nanofibers as high performance lithium ion battery anodes. Carbon.

[51] Zhang Y.-C., You Y., Xin S., Yin Y.-X., Zhang J., Wang P., Zheng X.-S., Cao F.-F., Guo Y.-G. (2016). Rice husk-derived hierarchical silicon/nitrogen-doped carbon/carbon nanotube spheres as low-cost and high-capacity anodes for lithium-ion batteries. Nano Energy.

[52] Xiao K., Ding L.-X., Chen H., Wang S., Lu X., Wang H. (2016). Nitrogen-doped porous carbon derived from residuary shaddock peel: A promising and sustainable anode for high energy density asymmetric supercapacitors. J. Mater. Chem. A.

[53] Sekar S., Lee Y., Kim D.Y., Lee S. (2019). Substantial LIB anode performance of graphitic carbon nanoflakes derived from biomass green-tea waste. Nanomaterials.

[54] Sekar S., Aqueel Ahmed A.T., Pawar S.M., Lee Y., Im H., Kim D.Y., Lee S. (2020). Enhanced water splitting performance of biomass activated carbon-anchored WO_3_ nanoflakes. Appl. Surf. Sci..

[55] Lee S., Kang T.W., Kim D.Y. (2005). Correlation of Magnetic Properties with Microstructural Properties for Columnar-Structured (Zn_1−x_Mn_x_)O/Al_2_O_3_ (0001) Thin Films. J. Cryst. Growth.

[56] Kaur N., Lee Y., Kim D.Y., Lee S. (2018). Optical bandgap tuning in nanocrystalline ZnO:Y films via forming defect-induced localized bands. Mater. Des..

[57] Lee S., Seong J., Kim D. (2010). Effects of laser-annealing using a KrF excimer laser on the surface, structural, optical, and electrical properties of AlZnO thin films. J. Korean Phys. Soc..

[58] Mishra P., Jain K.P. (2001). First- and second-order Raman scattering in nanocrystalline silicon. Phys. Rev. B Condens. Matter.

[59] Krause A., Tkacheva O., Omar A., Langklotz U., Giebeler L., Dörfler S., Fauth F., Mikolajick T., Weber W.M. (2019). In situ raman spectroscopy on silicon nanowire anodes integrated in lithium ion batteries. J. Electrochem. Soc..

[60] Sankar S., Ahmed A.T.A., Inamdar A.I., Im H., Im Y.B., Lee Y., Kim D.Y., Lee S. (2019). Biomass-derived ultrathin mesoporous graphitic carbon nanoflakes as stable electrode material for high-performance supercapacitors. Mater. Des..

[61] Sekar S., Kim D.Y., Lee S. (2020). Excellent Oxygen Evolution Reaction of Activated Carbon-Anchored NiO Nanotablets Prepared by Green Routes. Nanomaterials.

[62] Hung T.-F., Cheng W.-J., Chang W.-S., Yang C.-C., Shen C.-C., Kuo Y.-L. (2016). Ascorbic acid-assisted synthesis of mesoporous sodium vanadium phosphate nanoparticles with highly sp^2^-coordinated carbon coatings as efficient cathode materials for rechargeable sodium-ion batteries. Chem. Eur. J..

[63] Sekar S., Lee S., Vijayarengan P., Kalirajan K.M., Santhakumar T., Sekar S., Sadhasivam S. (2020). Upcycling of wastewater via effective photocatalytic hydrogen production using MnO_2_ nanoparticles-decorated activated carbon nanoflakes. Nanomaterials.

[64] Lee S., Lee Y., Kim S.M., Song E.B. (2018). Fully-Transparent Graphene Charge-Trap Memory Device with Large Memory Window and Long-Term Retention. Carbon.

[65] Ahmad W., Bahrani M.R.A., Yang Z., Khan J., Jing W., Jiang F., Chu L., Liu N., Li L., Gao Y. (2016). Extraction of nano-silicon with activated carbons simultaneously from rice husk and their synergistic catalytic effect in counter electrodes of dye-sensitized solar cells. Sci. Rep..

[66] Loaiza L.C., Monconduit L., Seznec V. (2019). Siloxene: A potential layered silicon intercalation anode for Na, Li and K ion batteries. J. Power Sources.

[67] Gönüllü Y., Kelm K., Mathur S., Saruhan B. (2014). Equivalent Circuit Models for Determination of the Relation between the Sensing Behavior and Properties of Undoped/Cr Doped TiO_2_ NTs. Chemosensors.

